# Identification and characterization of rabbit ROSA26 for gene knock-in and stable reporter gene expression

**DOI:** 10.1038/srep25161

**Published:** 2016-04-27

**Authors:** Dongshan Yang, Jun Song, Jifeng Zhang, Jie Xu, Tianqing Zhu, Zhong Wang, Liangxue Lai, Y. Eugene Chen

**Affiliations:** 1 Center for Advanced Models for Translational Sciences and Therapeutics, University of Michigan Medical Center, Ann Arbor, MI 48109, USA; 2 Department of Cardiac Surgery, Cardiovascular Research Center, University of Michigan, Ann Arbor, MI 48109, USA; 3 Guangzhou Institutes of Biomedicine and Health, Chinese Academy of Sciences, Guangzhou, 510530, China

## Abstract

The laboratory rabbit has been a valuable model system for human disease studies. To make the rabbit model more amendable to targeted gene knockin and stable gene over-expression, we identified a rabbit orthologue of the mouse Rosa26 locus through genomic sequence homology analysis. Real-time PCR and 5′ RACE and 3′ RACE experiments revealed that this locus encodes two transcript variants of a long noncoding RNA (lncRNA) (rbRosaV1 and rbRosaV2). Both variants are expressed ubiquitously and stably in different tissues. We next targeted the rabbit Rosa26 (rbRosa26) locus using CRISPR/Cas9 and produced two lines of knock-in rabbits (rbRosa26-EGFP, and rbRosa26-Cre-reporter). In both lines, all the founders and their offspring appear healthy and reproduce normally. In F1 generation animals, the rbRosa26-EGFP rabbits express EGFP, and the rbRosa26-Cre-reporter rabbits express tdTomato ubiquitously in all the tissues examined. Furthermore, disruption of rbRosa26 locus does not adversely impact the animal health and reproduction. Therefore, our work establishes rbRosa26 as a safe harbor suitable for nuclease mediated gene targeting. The addition of rbRosa26 to the tool box of transgenic research is expected to allow diverse genetic manipulations, including gain-of function, conditional knock out and lineage-tracing studies in rabbits.

The laboratory rabbit has been used as a classic model species for mammalian development and human disease studies^1^,^2^. Conventional pronuclear DNA microinjection has been the dominant method for generating transgenic rabbits, largely due to the lack of germline transmitting embryonic stem (ES) cells. It is known, however, that this method is associated with random insertion and uncontrollable copy number of the transgene. Recently, with the development of genome editing tools including ZFN, TALEN, and CRISPR/Cas9, it is possible to produce gene targeting transgenic (i.e. knock-out, knock-in) rabbits with high success rates. In 2012, we produced Apolipoprotein C-III Knock-out Rabbits using ZFN^3^. In 2014, we generated a number of gene knock-out rabbit lines using the CRISPR/Cas9 approach with high efficiencies^4^. In 2015, we reported high efficiency in producing knock-in rabbits through nuclease mediated homologous recombinations^5^. These novel knock-out and knock-in rabbit models are proven valuable for the study of a number of human diseases that currently are not well modeled by mice.

Rosa26 is a safe harbor locus broadly used for constitutive, ubiquitous gene expression in mice. It was first isolated in 1991 in a gene-trap mutagenesis screen of murine ES cells^6^. Knock-in of the transgene(s) to Rosa26 enables locus specific and copy number controlled transgenic, and is proven “safe” in mice because the transgene is adequately expressed without perturbing endogenous gene structure or function. To date, over 130 Rosa26 knock-in mouse lines have been created^7^.

The Rosa26 locus is conserved in a variety of species, including human, rats, and pigs^8^,^9^,^10^. It was not known, however, whether the Rosa26 locus exists in the rabbit genome, and if so, whether this locus possesses similar characteristics as shown in mice.

To address these questions, we identified and characterized the rabbit Rosa26 locus. We further conducted *in vivo* experiments to produce knock-in rabbits at this locus to functionally validate its applicability. Our results add rbRosa26 to the tool box of transgenic research. The availability of a safe harbor locus in this species is expected to allow diverse genetic manipulations, including gain-of function, loss-of-function and lineage-tracing studies in rabbits.

## Results

### Identification of mouse Rosa 26 orthologue in rabbit genome

**Figure 1:**
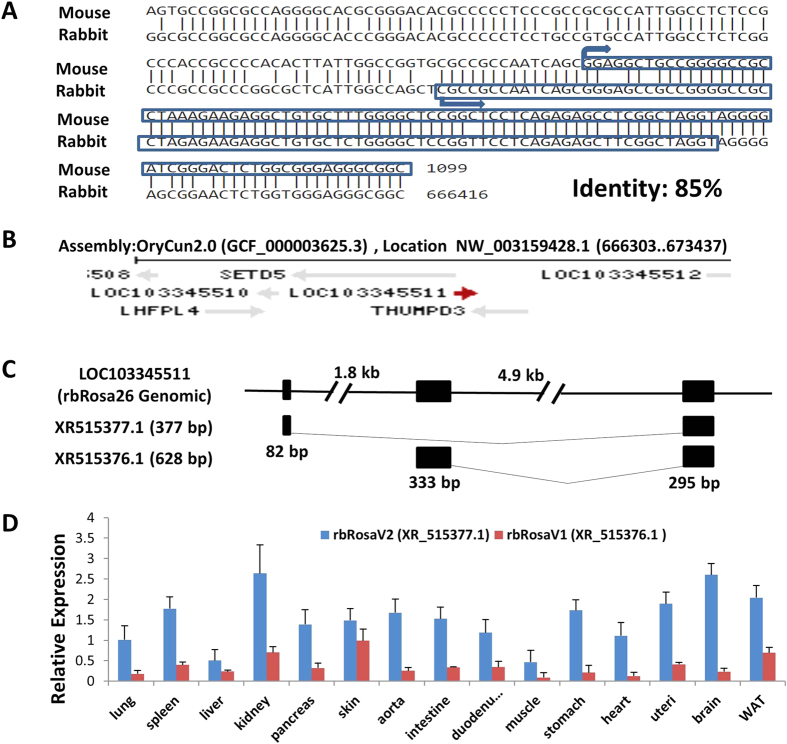
Identification of rabbit Rosa26 locus. **(A)** Alignment of the mouse Rosa26 sequence and rabbit locus 103345511 (GenBank: XR_515377.1), the putative rbRosa26 locus. Identity score 85%. (**B)** Illustration of genomic context of the rabbit locus 103345511 and its flanking regions (screen shot from NCBI website). (**C**) Schematic diagram of the rbRosa26 gene and 2 transcript variants. Top: rbRosa26 genomic sequence, comprising 3 exons (black boxes), and two introns. Bottom two rows: Two transcript variants rbRosa26V1 (628 bp, XR515376.1) and rbRosa26V2, (377 bp, XR515377.1). (**D)** Relative RNA expression (mean + SEM) of rbRosa26 transcripts in different adult rabbit tissues by real-time PCR. Data are normalized based on the average expression level of XR515377.1 in the lungs (Cq = 25.87). WAT: white adipose tissue.

Mouse, human, rat and pig data suggest that Rosa26 and its promoter region contained highly conserved sequences^8^,^9^,^10^. So we used the sequence of mouse Rosa26 exon1 (178 bp) plus promoter region (1 kb upstream of exon1) as a template to search the NCBI database by nucleotide blast against New Zealand rabbit reference genomic sequence (taxid: 9986), and identified a region with the highest degree of homology (Fig. 1A, 85% identity with region from 461 bp to 1099 bp) in the rabbit genome: uncharacterized locus LOC103345511 (Genomic Sequence: NW_003159428) (Fig. 1B). Genes flanking this locus are also the same to those in the mouse Rosa26 locus (SEDT5 and THUMPD3, Fig. 1B).

### RbRosa26 encodes two noncoding RNA variants

In mice, transcripts from the Rosa26 locus are ubiquitously expressed noncoding RNAs^11^. In rabbits, this putative rabbit Rosa26 locus, according to gene bank database, is predicted to transcribe two noncoding RNA variants (377 bp XR_515377.1 and 628 bp XR_515376.1, Fig. 1C). To verify this prediction, we designed primer sets based on the predicted transcripts sequences (Suppl. Table 1). Quantitative real-time PCR analysis demonstrated that these noncoding RNA variants were expressed in a wide variety of adult tissues (Fig. 1D). The slight variations of expression levels in different tissues are expected as this was also observed in the other species^8^,^9^,^10^. We performed the 5′ and 3′ RACE analysis and determined the full-length sequence of both variants, which confirmed the predicted transcripts (illustrated in Fig. 1C).

### RbRosa26 is amenable to nuclease mediated knock-in

We recently reported CRISPR/Cas9 mediated knock-in of EGFP to the rbRosa26 locus^5^. In this project, we used the same strategy to produce rbRosa26-Cre-reporter knock-in rabbits. Briefly, sgRNA targeting rbRosa26 is co-introduced to pronuclear stage rabbit embryos with Cas9 mRNA and the donor DNAs.

**Figure 2:**
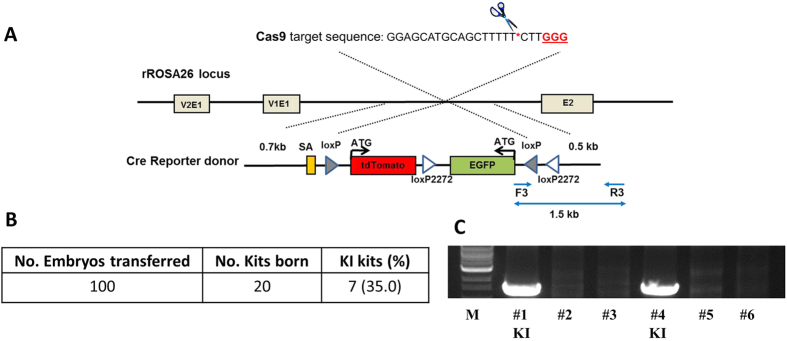
Production of rbRosa26-Cre-reporter rabbits. (**A**). Illustration of CRISPR/Cas9 mediated knock-in strategy to produce rbRosa26-Cre-reporter rabbits. (**B**) Summary of knock-in efficiencies. (**C**) Representative genotyping gel of rbRosa26-Cre-reporter founder (i.e. F0) rabbits. #s: # of corresponding F0 kit. KI: knock-in. M: NEB 1 kb DNA Ladder marker.

The donor DNA template (Fig. 2A) consists of a splice acceptor (SA), a promoterless tdTomato gene, and an inverted EGFP (iEGFP) flanked by homologous arms. To realize Cre-mediated removal of the tdTomato gene, inversion the expression of the iEGFP, and consequently the expression of EGFP, the loxP and mutant loxP2272 sites were arranged to flank the tdTomato and iEGFP genes (Fig. 2A). The heterotypic loxP sites in the knock-in vector would allow virtually any gene of interest be inserted into the rbRosa26 locus by Recombinase-mediated cassette exchange (RMCE).

We first determined the embryo development rates *in vitro* to see if adding donor DNA to the injection mixture would adversely affect embryo development. We injected 85 embryos, 43 developed to blastocysts stage. The rate (50.5%) is similar to the rates we achieved in Cas9 mediated knock-out work (34.2 to 68.4%), in which the injection mixture contains Cas9 mRNA and sgRNA but no donor DNA templates^4^.

After *in vitro* work, we conducted embryo transfer. The injected embryos were cultured overnight (20 hours) with 7.5 μM RS-1 to improve knock-in efficiency and then surgically transferred into the oviduct of synchronized recipient does. Out of 20 term kits, 7 (35%) were identified as founder knock-in animals based on PCR results (Fig. 2B,C). The knock-in efficiency is similar to that of the Rosa26-EGFP rabbit production. Such high knock-in rates indicate that rbRosa26 is highly amenable for nuclease mediated knock-in.

### Transgenes express ubiquitously at the rbRosa26 locus

**Figure 3:**
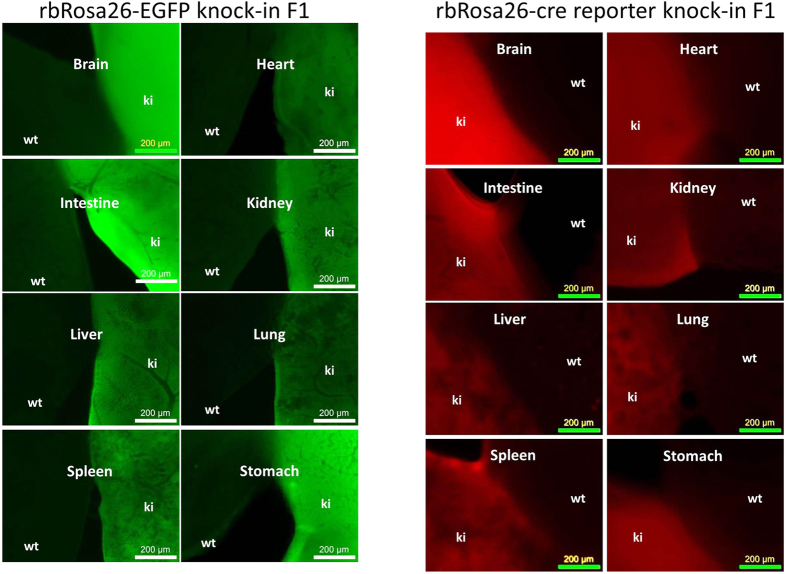
Transgene expression profiles at rbRosa26 locus. Left: EGFP expression in different organs/tissues in rbRosa26-EGFP rabbits. Right: tdTomato expression in different organs/tissues in rbRosa26-Cre-reporter rabbits. F1 generation rabbits were used for analysis in both lines.

We next examined the transgene profiles in rbRosa26-EGFP (Fig. 3, left panels) and rbRosa26-Cre-reporter (Fig. 3, right panels) rabbits. In both lines, EGFP and tdTomato were ubiquitously expressed in all organs (brain, heart, intestine, kidney, liver, lungs, spleen, and stomach) that were examined.

### Transgenes at the rbRosa26 can be stably passed to the next generations by germline transmission

To test germline transmission, founder rbRosa26-EGFP and rbRosa26-Cre-reporter rabbits were bred with wild type (WT) ones. Out of 8 F1 Rosa26-EGFP kits, 4 (50%) are confirmed positive for knock-in. Similarly, out of 7 F1 Rosa26-Cre-reporter kits, 4 (57%) are positive for knock-in. These results confirmed that transgene at the rbRosa26 locus can be transmitted to the germline.

### Disruption of rbRosa26 does not adversely affect health and reproduction

**Figure 4:**
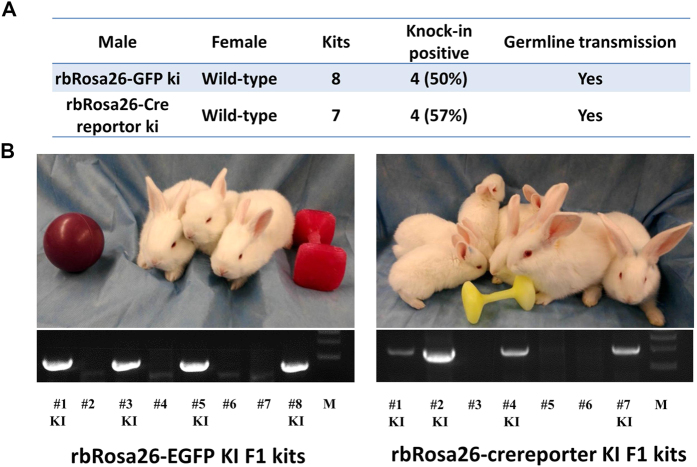
Germline transmission of rbRosa26 knock-in rabbit lines. (**A**) Summary of germline transmission rates of rbRosa26-EGFP and rbRosa26-Cre-reporter knock in founders. (**B**) Upper: Representative photos of F1 generation knock-in rabbits; lower: geneotyping gel of the F1 generation knock-in rabbits. #s: # of corresponding F1 kit. KI: knock-in. M. NEB 1 kb DNA Ladder marker.

In both lines, all founder and F1 knock-in rabbits appear healthy and undistinguishable for WT animals (Fig. 4B and Supp. Table 2). No abnormalities were observed in development and reproduction, indicating that disruption of rbRosa26 locus does not adversely affect animal health and reproduction, validating the safe harbor status of this locus.

### Cre mediated recombination

**Figure 5:**
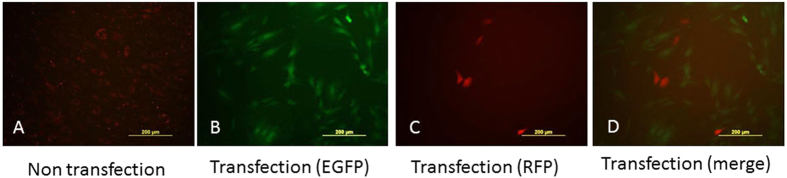
Cre mediated recombination in rbRosa26-Cre-reporter cells. (**A**) Without Cre, rbRosa26-Cre-reporter cells express tdTomato (red). (**B**) RbRosa26-Cre-reporter cells switch to express EGFP (green) as a result of Cre mediated recombination after successful transfection of pBS185CMV-Cre plasmid. (**C**) Some residual rbRosa26-Cre-reporter cells still expressing tdTomato after Cre transfection. (**D**) Merged image of (**B**,**C**).

To test this Cre reporter rabbit line, a Cre expression plasmid was transfected into skin fibroblast cells isolated from one rbRosa26-Cre-reporter knock-in rabbit. Forty-eight hours later, GFP or RFP expression were determined by epifluorescent microscopy. As expected, most of the transfected cells were EGFP positive and RFP negative, in comparison, all cells were RFP positive and EGFP negative in the untransfected cell culture (Fig. 5). These results indicated that this rabbit Cre reporter line we have established can report the expression of Cre recombinase efficiently.

### Off target detection

We examined off target effects in the KI founders, following the strategies described in our previous paper^4^. We used BLASTn to identify exact match to the 15 nt sequence (12 nt seed region and 3 nt NGG), and found a total of 24 potential off target loci, of which one is within an exon region of gene Has2 (Supp. Fig. 1A). Considering the fact that mutations in an exon region are more likely to cause gene mutation and phenotype changes in the animals, we looked at this locus in-four breeding founders using T7 endonuclease assay. None of the 4 founders tested contained mutations in this exon region (Supp. Fig. 1B).

## Discussion

Since the first transgenic rabbit produced by Hammer and his colleagues^12^, transgenic rabbit models were generated by different laboratories worldwide for different research purposes, such as studies of lipid metabolism and atherosclerosis^13^,^14^,^15^,^16^,^17^,^18^, oncology^19^,^20^, acquired immunodeficiency syndrome (AIDS)^21^,^22^,^23^, and bioreactors for the production of pharmaceutical proteins^24^,^25^, However most of these rabbit models are produced by pronuclear microinjection of foreign DNA, in which the foreign genes are randomly inserted in the rabbit genome. This may be problematic as transgenes introduced into random sites within the rabbit genome are often subjected to position effects, including variations in the expression levels of the transgene caused by variation in the copy number of the transgenes or lack of crucial regulator elements related to the integration site, and potentially disrupting endogenous gene function through insertional mutagenesis. Therefore, researchers often produce multiple lines for screening of lines with adequate transgene expression and for obtaining reproducible results.

The rbRosa26 locus characterized here is the first transgene safe harbor identified in the rabbit genome. Similar to that in mouse, rat, human and pig^8^,^9^,^10^,^11^, the rbRosa26 locus encodes long noncoding RNAs in a broad spectrum of tissues , a promoterless transgene integrated to this locus can be expressed universally, and disruption of the rbRosa26 locus does not lead to any abnormality based on our observations. These features define rbRosa26 a safe harbor locus, and makes it an attractive locus for versatile transgenic applications, including gain-of function, loss-of-function and lineage-tracing studies in rabbit.

Moreover, the rbRosa26 locus appears to be highly amenable for nuclease mediated knock-in. Greater than 20% knock-in rates were achieved in both lines attempted in the present study. Hence the transgene safe harbor identified in this study in combination with the emerging gene editing nuclease tools such as CRISPR/Cas9 enabled efficient direct targeted integration of single copy (or any desired number) transgene with predictable transgene expression, without damaging any endogenous genes.

Two novel transgenic rabbit lines were produced in this present study. The fidelity of EGFP expression in the rbRosa26-EGFP line makes it an excellent marker line for transplantation or chimera experiments. The Cre reporter rabbit line is useful for tissue specific transgenic studies. In addition, priming of the Cre reporter rabbit line with a loxP/mutant loxP2272 homing site created a valuable model in which any genetic material of interest can be efficiently introduced using Recombinase-mediated cassette exchange (RMCE)^10^,^26^, as an alternative method for targeted integration in this locus.

In summary, the present work identifies a safe harbor locus, rbRosa26, in the rabbit genome. The rbRosa26 encodes two ubiquitously expressed noncoding RNA variants, and is amenable to nuclease mediated knock-in. Disruption of the rbRosa26 does not lead to abnormality in animal health and reproduction. We expect that using rbRosa26 for transgenic applications will facilitate the development of rabbit models for translational sciences and therapeutics.

## Materials and Methods

### Animals

The rabbits used in this work were from the New Zealand White (NZW) strain. All animal maintenance, care and use procedures were reviewed and approved by the University Committee on the Use and Care of Animals of the University of Michigan. All efforts were made to minimize suffering. All the methods were carried out in accordance with the approved guidelines.

### rbRosa26 locus identification

The sequence of mouse Rosa26 Promoter region plus exon1(1178 bp) was blasted against the New Zealand rabbit (taxid:9986) reference genomic sequence using nucleotide blast. For analysis by quantitative PCR (qPCR) and RT-PCR, total RNA from lung, spleen, liver, kidney, pancreas, skin, aorta, intestine, duodenum, skeletal muscle, stomach, heart, uteri, brain and white adipose tissue (WAT) was isolated from wild-type NZW rabbit using the RNeasy kit (Qiagen). Reverse transcription was used to generate cDNA (SuperScript® III First-Strand Synthesis System, Thermo Fisher Scientific, 18080–05). For qPCR analysis, samples were analyzed on a BioRad CFX Connect™ Real-Time PCR Detection System and amplification was detected using the SYBR green method (BioRad, iQ SYBR green supermix). PCR primers are as following: F1: 5′- agaagaggctgtgctctgg -3′, R1: 5′- cagtcaagtgtcgtcccact -3′; F2: 5′- aggctggcctcaactttgta -3′; R2: 5′- acagccagtcaagtgtcgtc-3′.

### 5′, 3′ rapid amplification of cDNA ends analysis and identification of full-length noncoding RNA

5′ and 3′ rapid amplification of cDNA ends (RACE) was performed using the SMARTer® RACE 5′/3′ Kit (Clontech, 634858), according to the manufacturer’s protocol. 5′-RACE-Ready cDNA and 3′-RACE-Ready cDNA were Synthesized using a 5′-CDS Primer A or a 3′-CDS Primer A (included in the kit) respectively, and the cDNAs were amplified using an universal primer A mix with 5′ or 3′ gene specific primers (GSP, 5′-GSP: 5′-GATTACGCCAAGCTTacagccagtcaagtgtcgtcccactcaa-3′, 3′-GSP for transcript variant 2: 5′-GATTACGCCAAGCTTggctgtgctctggggctccggttcctca-3′ and 3′-GSP for transcript variant 1: 5′-GATTACGCCAAGCTTattcttcgggccatcctgtattgctgttag-3′). Amplified cDNAs were cloned into the pRACE vector using the included In-Fusion HD Cloning Kit, and sequenced.

### CRISPR/Cas9 plasmids construction and RNA synthesis

For CRISPR/Cas9 mediated knock-in experiments, the Cas9 expression plasmid JDS246 and sgRNA expression plasmid DR274 were obtained from Addgene. sgRNA was designed using Zifit software (http://zifit.partners.org/ZiFiT/), synthesized and cloned into the plasmid DR274. The targeted sequence for rbRosa26 are shown in Fig. 2A.

Cas9 mRNAs were transcribed *in vitro*, capped and polyadenylated using the T7 mScript™ Standard mRNA Production System (C-MSC100625, CELLSCRIPT, Madison, WI). sgRNA was *in vitro* transcribed by using T7-Scribe™ Standard RNA IVT Kit (C-AS3107, CELLSCRIPT). Cas9 mRNA and sgRNA were diluted in RNase-free TE buffer (1 mM Tris-Cl pH 8.0, 0.1 mM EDTA), stored in −80 °C in 10 μl aliquots, and were thawed and kept on ice before microinjection.

### Construction of homologous recombination donor vectors

HR donor vectors (Supp. Fig. 2) were constructed by standard molecular-cloning methods. For EGFP knock-in, the donor vector consists of the 0.7-kb 5′ homology arm and the 0.5-kb 3′ homology arm, flanking an adenoviral splice acceptor sequence, followed by the 2-kb EGFP expression cassette. For a Cre reporter knock in, a splice acceptor (SA), a promoterless tdTomato gene, and an inverted EGFP (iEGFP) gene was inserted between the homologous arms. The loxP and mutant loxP2272 sites were arranged to flank the tdTomato and iEGFP genes. (Fig. 2A)

### Microinjection and embryo transfer

Sexually matured (6–18 months) New Zealand White (NZW) female rabbits were superovulated by subcutaneous injection of follicle-stimulating hormone (FSH, Folltropin-V, Bioniche Life Sciences, Canada) twice/day with a dosage of 3 mg for the first two injections, 5 mg for the next two injections and 6 mg for the last two injections. Seventy-two hours after the first FSH injection, a single intravenously injection of 200 IU human chorionic gonadotropin (hCG, Chorulon, Intervet, Holland) was administered to induce ovulation. The superovulated females were mated with male rabbit immediately after hCG injection. Sexually matured recipient female rabbits were synchronized by stimulate mechanically in the vagina and injection of 0.3 ml Gonadotropin-releasing hormone (GnRH) agonist (Receptal®, Merck animal health) intramuscularly. Eighteen hours post insemination, the superovulated rabbits were euthanized. The oviduct ampullae were recovered, flushed with 10 ml of Hepes buffered manipulation (HM) medium containing 25 mM Hepes buffered TCM 199 (#12350039, Life Technologies, Grand Island, NY) supplemented with 10% fetal bovine serum (FBS, #12003C, Sigma, St. Louis, MO), and the recovered oocytes were observed under a microscope for the occurrence of fertilization, and then kept in the HM at 38.5 °C in air.

Microinjection was performed on pronuclear stage embryos 19–21 h post insemination using a micromanipulator under the inverted microscope equipped with a differential interference contrast (DIC) device. Rabbit embryo was held with a holding glass pipette (120–150 μm diameter) in HM medium. A mixture containing 100 ng/μl donor DNA, 150 ng/μl Cas9 mRNA and 6 ng/μl sgRNA were used for cytoplasm microinjection. Approximately 2–5 pL mixture was injected to each embryo. Injected embryos were washed three times in embryo culture medium, which consisted of Earle’s Balanced Salt Solution (E2888, Sigma) supplemented with non-essential amino acids (M7145, Sigma), essential amino acids (B-6766, Sigma), 1 mM L-glutamine (25030-081, Life Technologies), 0.4 mM sodium pyruvate (11360-070, Life Technologies) and 10% FBS. The injected embryos were cultured overnight (20 hours) with 7.5 μM RS-1 treatment before surgically transferred into the oviduct of a synchronized recipient doe. Twenty to thirty embryos were transferred to one recipient doe. For *in vitro* validation, instead of transferring to recipient doe, the injected and treated embryos were washed and cultured in medium for additional 2–3 days until they reach blastocyst stage.

### Confirmation of gene targeting events

For *in vitro* validation, blastocyst stage embryos were lysed individually and genomic DNA extracted. Before PCR reaction, the whole genome was replicated using a REPLI-g® Mini Kit (Qiagen, Germantown, MD) following the manufacturer’s protocol with slight modification. Briefly, for harvesting denatured DNA, 3.5 μl Buffer D2 was added to each embryo, mixed by vortexing and centrifuged briefly. The samples were incubated on ice for 10 min. After that 3.5 μl Stop Solution was added, mixed by vortexing and centrifuged briefly. For replication, 2 μl of the denatured DNAs were added to 8 μl master mix and incubate at 30 °C for 10–16 h. Then REPLI-g Mini DNA Polymerase was inactivated by heating at 65 °C for 3 min. To determine genotypes of founder animals, ear skin tissues were biopsied, and genomic DNA extracted.

Genomic DNA were next used for PCR using corresponding primers (Primers for indel detection: forward 5′-agccctaaattcaagccctgtg-3′, reverse: 5′-agaagcctgtcccctaaacta-3′, sequence primer: 5′-ggggagtgaaccagcagacg-3′. Primers for Rosa-EGFP ki detection: forward 5′-ctccccagcaggcagaagt-3′, reverse 5′-aaggtgagaaacaggcagaaatagt-3′. Primers for Rosa-Cre reporter ki detection: forward 5′-agaagcctgtcccctaaacta-3′, reverse 5′-cggcggcggtcacgaactcc-3′). PCR products were analyzed with agarose gel electrophoresis for knock-in detection or purified and sequenced for detection of indel mutations. A knock-in event will show a 0.8 kb band for rbRosa-EGFP and 1.5 kb band for Cre reporter knock in. For indel detection, PCR products were sequenced. On the chromatographic curves, peaks on peaks approximate the targeting site indicate an indel event.

### Fibroblast cell isolation and electroporation

Rabbit ear fibroblasts (REFs) were isolated from ear skin tissues of the newborn Kits. Ear tissues were washed with PBS and hair removed, immerged in 75% ethanol for 5 min and washed with PBS containing 2% penicillin-streptomycin. The ear tissues were then minced, and digested by collagenase-DNase in Dulbeccos’ modified Eagle’s medium (DMEM) supplemented with 10% fetal bovine serum (FBS) (Sigma), 2% penicillin-streptomycin (Thermo), 1 mg/ml Collagenase IV (Life Technology), and 100 KU/ml DNaseI (Sigma) for 4–5 h at 37 °C. Dissociated cells were centrifuged at 250 g for 5 min, followed by suspension in culture medium (DMEM supplemented with 10% FBS, 0.5% penicillin-streptomycin, 1% Non-Essential Amino Acids, 2 mM GlutaMAX, and 1 mM sodium pyruvate). The cells were plated on 10 cm culture dishes, cultured and frozen in FBS containing 10% dimethyl sulfoxide for future use.

For Cre expressing plasmid transfection, REF cells were thawed and plated on 6-well plates. After 12–24 hours, Lipofectamine® 2000 mixed with plasmid DNA were added (2.5 ug DNA, 10 ul Lipofectamine® 2000 in 250 ul culture medium) according to the manufacturer’s protocol. Fourty eight hours later, cells were detected under fluorescence microscope.

## Additional Information

**How to cite this article**: Yang, D. *et al.* Identification and characterization of rabbit ROSA26 for gene knock-in and stable reporter gene expression. *Sci. Rep.* **6**, 25161; doi: 10.1038/srep25161 (2016).

## Supplementary Material

Supplementary Information
