# Supplementary material for: Identification and characterization of rabbit ROSA26 for gene knock-in and stable reporter gene expression
Source: Sci Rep. 2016 Apr 27;6:25161. doi: 10.1038/srep25161 (PMC4846827; doi:10.1038/srep25161)
Supplement: Supplementary Information [file srep25161-s1.pdf]

Supplementary data

Title:

Identification and characterization of rabbit ROSA26 for gene knock-in and stable reporter gene expression

Authors:

Dongshan Yang[1], Jun Song[1], Jifeng Zhang[1], Jie Xu[1], Tianqing Zhu[1], Zhong Wang[2], Liangxue Lai[3], Y.Eugene Chen[1,2*]

Affiliations:

1. Center for Advanced Models for Translational Sciences and Therapeutics, University of Michigan Medical Center, Ann Arbor, MI 48109,USA

2. Department of Cardiac Surgery, Cardiovascular Research Center, University of Michigan, Ann Arbor, MI 48109, USA

3. Guangzhou Institutes of Biomedicine and Health, Chinese Academy of Sciences, Guangzhou, 510530, China

*Correspondence should be addressed to echenum@med.umich.edu

Supp. Table 1. Primers used in the present study.

	**SIZE**	**sequences**
For indel detection	F1/R1, 2kb	F1: 5’- agccctaaattcaagccctgtg-3’<br>R1: 5’- agaagcctgtcccctaaacta-3’<br>Pseq: 5’- ggggagtgaaccagcagacg-3’
For Rosa-EGFP ki detection	F2/R2, 0.8 kb	F2: 5’- ctccccagcaggcagaagt-3’<br>R2: 5’- aaggtgagaaacaggcagaaatagt-3’
For Rosa-Cre reporter ki detection	F3/R3, 1.5 kb	F3: 5’-agaagcctgtcccctaaacta-3’<br>R3: 5’-cggcggcggtcacgaactcc-3’
5’-GSP		5'-gattacgccaagctttcaggttcagggggaggtgtgggaggtt-3’
3’-GSPs		5’-gattacgccaagcttggctgtgctctggggctccggttcctca-3’<br>5’- gattacgccaagcttattcttcgggccatcctgtattgctgttag-3’
For Realtime-PCR	F4/R4, 66 bp<br><br>F5/R5, 79 bp	F4: 5’- agaagaggctgtgctctgg -3’<br>R4: 5’- cagtcaagtgtcgtcccact -3’<br><br>F5: 5’- aggctggcctcaactttgta -3’<br>R5: 5’- acagccagtcaagtgtcgtc-3’

Supp. Table 2. Weight comparison of rbRosa26-GFP and rbRosa26-Cre-reporter knock-in rabbits (F1 generation) with their age matched controls.

	**Weight (kg) ± SD (n)**		
**Age**	**rbRosa26-GFP ki**	**rbRosa26-Cre reporter ki**	**WT**
5 months	3.27 ± 0.26 (9)		3.29 ± 0.07 (4)
4 months		2.70 ± 0.04 (5)	2.79 ± 0.10 (4)

Supp. Fig. 1. Off target analysis of in founder rabbits. (A) Only one off target match is identified to be within an exon region of a gene. It is in the 3' UTR of Has2 gene. Red characters are the seed + PAM sequence in the sgRNA. (B) T7 endonuclease I assay revealed gene editing events in the on target region but none at the off target region in all founder animals examined. wt: wild type. M: NEB 100 bp DNA Ladder marker. On target: samples are from the on target region (i.e. rbRosa26). Off target: samples are from the off target regions (3' UTR of Has2) from founder #1 to #4. #1 and #2: breeding founders of rbRosa26-GFP knock-in rabbits. #3 and #4: breeding founders of rbRosa26-Cre-reporter knock-in rabbits.

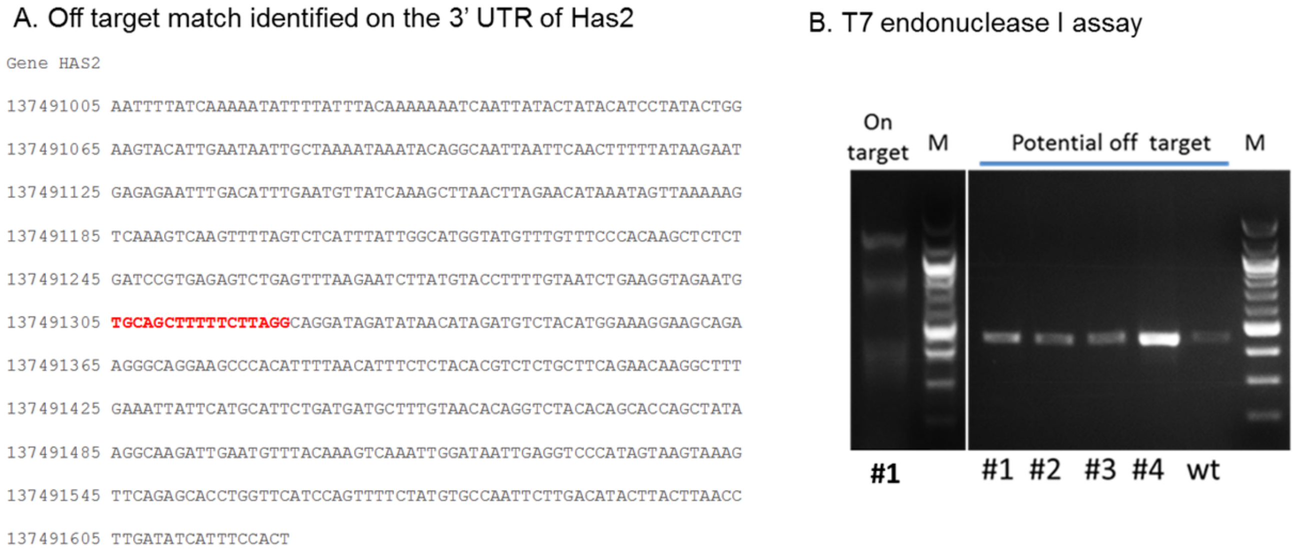

Supp. Fig. 2. Donor DNA sequence and annotation

<u>rbRosa26-EGFP donor DNA sequence:</u>

5’-

acaatgcccatgccttcaactgtgtgtgtgtttttttttttataagacttttaaaagtctgatgggaaagacaccccaaggacagtattcttcagag

cttcggtgttcagagagagctctcagacagttgcctttctatataatacaaattctcaatcccacatccagagacgataaattcacagtgtgtgtgt

Homologous Arm

ggcatccccaggggcaagcgctagcattctgcatcttttcaagtgctgtgtctgtActgcagagtagctgcagtgaggtgctaggcctgttttcct

gacagaaatggttacaacttcatagaaactatgctttttggccctcagtttttcaccaactgaaatattttcatttttccaaaaacaggcagtggg

aaaacattgaaaagcaaggattttgtttaattctttttgagactctcttttaaaaactttttaaaaatactttaaaaaatgcttttaaattgcttttt

tgaacaagaaaaatttgtgtgatagtatgaatctaagttacactccttaaggacatggtgatttaattctttgggtctaggcacagctgggagttt

aataaatgttgcttttgtgaaattggtttgtgaatagtcatttaaacccacaaaacatagaaggaaggttcagctcatggaggaacttggagtc

*atacttaagtcgcctgatattctattcatagatgaaactcggcctcatggtctcga*GGCTAGCAGATCTGTAGGGCGCAGTAGTCCA

Splicing Acceptor

GGGTTTCCTTGATGATGTCATACTTATCCTGTCCCTTTTTTTTCCACAGCTCGCGGTTGAGGACAAACTCTTCGCGGT

CTTTCCAGTGGGGATCGACGGTATCGATAAGCTTGGTACCGCGGGCCCGGGATCCACCGGTCGCCACCATGGTGA

GCAAGGGCGAGGAGCTGTTCACCGGGGTGGTGCCCATCCTGGTCGAGCTGGACGGCGACGTAAACGGCCACAAG

TTCAGCGTGTCCGGCGAGGGCGAGGGCGATGCCACCTACGGCAAGCTGACCCTGAAGTTCATCTGCACCACCGGC

EGFP cDNA

AAGCTGCCCGTGCCCTGGCCCACCCTCGTGACCACCCTGACCTACGGCGTGCAGTGCTTCAGCCGCTACCCCGACC

ACATGAAGCAGCACGACTTCTTCAAGTCCGCCATGCCCGAAGGCTACGTCCAGGAGCGCACCATCTTCTTCAAGGA

CGACGGCAACTACAAGACCCGCGCCGAGGTGAAGTTCGAGGGCGACACCCTGGTGAACCGCATCGAGCTGAAGG

GCATCGACTTCAAGGAGGACGGCAACATCCTGGGGCACAAGCTGGAGTACAACTACAACAGCCACAACGTCTATA

TCATGGCCGACAAGCAGAAGAACGGCATCAAGGTGAACTTCAAGATCCGCCACAACATCGAGGACGGCAGCGTG

CAGCTCGCCGACCACTACCAGCAGAACACCCCCATCGGCGACGGCCCCGTGCTGCTGCCCGACAACCACTACCTGA

GCACCCAGTCCGCCCTGAGCAAAGACCCCAACGAGAAGCGCGATCACATGGTCCTGCTGGAGTTCGTGACCGCCG

CCGGGATCACTCTCGGCATGGACGAGCTGTACAAGTAAAGCGGCCGCGACTCTAGATCATAATCAGCCATACCAC

ATTTGTAGAGGTTTTACTTGCTTTAAAAAACCTCCCACACCTCCCCCTGAACCTGAAACATAAAATGAATGCAATTG
TTGTTGTTAACTTGTTTATTGCAGCTTATAATGGTTACAAATAAAGCAATAGCATCACAAATTTCACAAATAAAGCA
TTTTTTTCACTGCATTCTAGTTGTGGTTTGTCCAAACTCATCAATGTATCTTAAGGCGTAAATTGTAAGCGTTAATAT
TTTGTTAAAATTCGCGTTAAATTTTTGTTAAATCAGCTCATTTTTTAACCAATAGGCCGAAATCGGCAAAATCCCTTA
TAAATCAAAAGAATAGACCGAGATAGGGTTGAGTGTTGTTCCAGTTTGGAACAAGAGTCCACTATTAAAGAACGT
GGACTCCAACGTCAAAGGGCGAAAAACCGTCTATCAGGGCGATGGCCCACTACGTGAACCATCACCCTAATCAAG
TTTTTTGGGGTCGAGGTGCCGTAAAGCACTAAATCGGAACCCTAAAGGGAGCCCCCGATTTAGAGCTTGACGGGG
AAAGCCGGCGAACGTGGCGAGAAAGGAAGGGAAGAAAGCGAAAGGAGCGGGCGCTAGGGCGCTGGCAAGTGT
AGCGGTCACGCTGCGCGTAACCACCACACCCGCCGCGCTTAATGCGCCGCTACAGGGCGCGTCAGGTGGCACTTT
TCGGGGAAATGTGCGCGGAACCCCTATTTGTTTATTTTTCTAAATACATTCAAATATGTATCCGCTCATGAGACAAT
AACCCTGATAAATGCTTCAATAATATTGAAAAAGGAAGAGTCCTGAGGCGGAAAGAACCAGCTGTGGAATGTGTG
TCAGTTAGGGTGTGGAAAGTCCCCAGGCTCCCCAGCAGGCAGAAGTATGCAAAGCATGCGGTA*cctgttctaaccatc*
actgtaatcttctgtgcttgacttccttgagctttttgtcttaaataaaccagctacctctgtttgtgaactcctgcatactgtttcttagaggacaga
aattcattatattaaacgtttgtacttgaatgaacaagttaggatgctttaaaaaaatctatacatttgaaaagtcagagttacaccaagagcgt
aggagaggcagagagagagaggtctcccgtctgctggttcactccccagttggccgcaagggctggagctgcaccagtcagaagccagaagc
tttttccaggtctctgatgtgggtgcatgggcacaaggacttgcactgtcttctactgctttcctaggccataggagagctggattggaagtggag
gaaccgggactcgaactgttgtacatagggatgtcagtattgcaggcagcagctttacccactaacccacagtgctggccctgactcttgttaag
Homologous Arm
ttaccaatccaggagtgtggtacttgactgtgcaggttgag-3’

<u>rbRosa26-cre-reporter donor DNA sequence:</u>

5’-
acaatgcccatgccttcaactgtgtgtgtgtttttttttttataagacttttaaaagtctgatgggaaagacaccccaaggacagtattcttcagag
Homologous Arm

cttcggtgttcagagagagctctcagacagttgcctttctgtgtagtgcaggttctcagtcccacatccagagacgataaattcacagtgtgtgtgt

ggcatccccaggggcaagcgctagcattctgcatcttttcaagtgctgtgtctgtActgcagagtagctgcagtgaggtgctaggcctgttttcct

gacagaaatggttacaacttcatagaaactatgctttttggccctcagtttttcaccaactgaaatattttcatttttccaaaaacaggcagtggg

aaaacattgaaaagcaaggattttgtttaattctttttgagactctcttttaaaaactttttaaaaatactttaaaaaatgcttttaaattgcttttt

tgaacaagaaaaatttgtgtgatagtatgaatctaagttacactccttaaggacatggtgatttaattctttgggtctaggcacagctgggagttt

aataaatgttgcttttgtgaaattggtttgtgaatagtcatttaaacccacaaaacatagaaggaaggttcagctcatggaggaacttggagtc

*atacttaagtcgcctgatattctattcatagatgaaactcgggcctcatggtctcga*GGCTAGCAGATCTAATTGGCGGAAGGCCGTC

AAGGCCTAGGCGCGCCTCAGTACTGCCTGCAGGGTGACCTGCACGTCTAGGGCGCAGTAGTCCAGGGTTTCCTTG

Splicing Acceptor

ATGATGTCATACTTATCCTGTCCCTTTTTTTTCCACAGCTCGCGGTTGAGGACAAACTCTTCGCGGTCTTTCCAGTAA

LoxP

GAATTCCTCGATCGAGGGACCTAATAACTTCGTATAGCATACATTATACGAAGTTATATTAAGGGTTCCGCAAGCT

TTCGCCACCATGGTGAGCAAGGGCGAGGAGGTCATCAAAGAGTTCATGCGCTTCAAGGTGCGCATGGAGGGCTC

TdTomato cDNA

CATGAACGGCCACGAGTTCGAGATCGAGGGCGAGGGCGAGGGCCGCCCCTACGAGGGCACCCAGACCGCCAAG

CTGAAGGTGACCAAGGGCGGCCCCCTGCCCTTCGCCTGGGACATCCTGTCCCCCCAGTTCATGTACGGCTCCAAGG

CGTACGTGAAGCACCCCGCCGACATCCCCGATTACAAGAAGCTGTCCTTCCCCGAGGGCTTCAAGTGGGAGCGCG

TGATGAACTTCGAGGACGGCGGTCTGGTGACCGTGACCCAGGACTCCTCCCTGCAGGACGGCACGCTGATCTACA

AGGTGAAGATGCGCGGCACCAACTTCCCCCCCGACGGCCCCGTAATGCAGAAGAAGACCATGGGCTGGGAGGCC

TCCACCGAGCGCCTGTACCCCCGCGACGGCGTGCTGAAGGGCGAGATCCACCAGGCCCTGAAGCTGAAGGACGG

CGGCCACTACCTGGTGGAGTTCAAGACCATCTACATGGCCAAGAAGCCCGTGCAACTGCCCGGCTACTACTACGT

GGACACCAAGCTGGACATCACCTCCCACAACGAGGACTACACCATCGTGGAACAGTACGAGCGCTCCGAGGGCCG

CCACCACCTGTTCCTGGGGCATGGCACCGGCAGCACCGGCAGCGGCAGCTCCGGCACCGCCTCCTCCGAGGACAA

CAACATGGCCGTCATCAAAGAGTTCATGCGCTTCAAGGTGCGCATGGAGGGCTCCATGAACGGCCACGAGTTCGA

GATCGAGGGCGAGGGCGAGGGCCGCCCCTACGAGGGCACCCAGACCGCCAAGCTGAAGGTGACCAAGGGCGGC

CCCCTGCCCTTCGCCTGGGACATCCTGTCCCCCCAGTTCATGTACGGCTCCAAGGCGTACGTGAAGCACCCCGCCG

ACATCCCCGATTACAAGAAGCTGTCCTTCCCCGAGGGCTTCAAGTGGGAGCGCGTGATGAACTTCGAGGACGGCG

GTCTGGTGACCGTGACCCAGGACTCCTCCCTGCAGGACGGCACGCTGATCTACAAGGTGAAGATGCGCGGCACCA

ACTTCCCCCCCGACGGCCCCGTAATGCAGAAGAAGACCATGGGCTGGGAGGCCTCCACCGAGCGCCTGTACCCCC

GCGACGGCGTGCTGAAGGGCGAGATCCACCAGGCCCTGAAGCTGAAGGACGGCGGCCACTACCTGGTGGAGTTC

AAGACCATCTACATGGCCAAGAAGCCCGTGCAACTGCCCGGCTACTACTACGTGGACACCAAGCTGGACATCACCT

CCCACAACGAGGACTACACCATCGTGGAACAGTACGAGCGCTCCGAGGGCCGCCACCACCTGTTCCTGTACGGCA

TGGACGAGCTGTACAAGTAGGCGGCCGCGACTCTAGATCATAATCAGCCATACCACATTTGTAGAGGTTTTACTTG

CTTTAAAAAACCTCCCACACCTCCCCCTGAACCTGAAACATAAAATGAATGCAATTGTTGTTGTTAACTTGTTTATTG

CAGCTTATAATGGTTACAAATAAAGCAATAGCATCACAAATTTCACAAATAAAGCATTTTTTTCACTGCATTCTAGTT

LoxP2272

GTGGTTTGTCCAAACTCATCAATGGATCCATAACTTCGTATAGGATACTTTATACGAAGTTATTTGGTACCAAACCA

CAACTAGAATGCAGTGAAAAAAATGCTTTATTTGTGAAATTTGTGATGCTATTGCTTTATTTGTAACCATTATAAGC

TGCAATAAACAAGTTAACAACAACAATTGCATTCATTTTATGTTTCAGGTTCAGGGGGAGGTGTGGGAGGTTTTTT

AAAGCAAGTAAAACCTCTACAAATGTGGTATGGCTGATTATGATCTAGAGTCGCGGCCGCTTTACTTGTACAGCTC

GTCCATGCCGAGAGTGATCCCGGCGGCGGTCACGAACTCCAGCAGGACCATGTGATCGCGCTTCTCGTTGGGGTC

TTTGCTCAGGGCGGACTGGGTGCTCAGGTAGTGGTTGTCGGGCAGCAGCACGGGGCCGTCGCCGATGGGGGTGT

TCTGCTGGTAGTGGTCGGCGAGCTGCACGCTGCCGTCCTCGATGTTGTGGCGGATCTTGAAGTTCACCTTGATGCC

GTTCTTCTGCTTGTCGGCCATGATATAGACGTTGTGGCTGTTGTAGTTGTACTCCAGCTTGTGCCCCAGGATGTTGC

CGTCCTCCTTGAAGTCGATGCCCTTCAGCTCGATGCGGTTCACCAGGGTGTCGCCCTCGAACTTCACCTCGGCGCG

GGTCTTGTAGTTGCCGTCGTCCTTGAAGAAGATGGTGCGCTCCTGGACGTAGCCTTCGGGCATGGCGGACTTGAA

GAAGTCGTGCTGCTTCATGTGGTCGGGGTAGCGGCTGAAGCACTGCACGCCGTAGGTCAGGGTGGTCACGAGGG

EGFP cDNA

TGGGCCAGGGCACGGGCAGCTTGCCGGTGGTGCAGATGAACTTCAGGGTCAGCTTGCCGTAGGTGGCATCGCCC

TCGCCCTCGCCGGACACGCTGAACTTGTGGCCGTTTACGTCGCCGTCCAGCTCGACCAGGATGGGCACCACCCCG

LoxP

GTGAACAGCTCCTCGCCCTTGCTCACCATGGTGGATCTATAACTTCGTATAATGTATGCTATACGAAGTTATCCTGC

ATCGACCTAGACTAGCTAGAACTAGTATAACTTCGTATAAAGTATCCTATACGAAGTTATCCTGCAGGGGATTTAA

LoxP2272

TTAACTGGCCTCATGGGCCTTCCGCTCACTGCCCGCTTTCCAGTCGGGAAACCTGTCGTGCCAGCTGCATTAACATG

GTCATAGCTGTTTGCATGCCGGTA*cctgttctaaccatcactgtaatcttctgtgcttgacttccttgagctttttgtcttaaataaaccagc*

Homologous Arm

tacctctgtttgtgaactcctgcatactgtttcttagaggacagaaattcattatattaaacgtttgtacttgaatgaacaagttaggatgctttaaa

aaaatctatacatttgaaaagtcagagttacaccaagagcgtaggagaggcagagagagagaggtctcccgtctgctggttcactccccagtt

ggccgcaagggctggagctgcaccagtcagaagccagaagctttttccaggtctctgatgtgggtgcatgggcacaaggacttgcactgtcttc

tactgctttcctaggccataggagagctggattggaagtggaggaaccgggactcgaactgttgtacatagggatgtcagtattgcaggcagc

agctttacccactaacccacagtgctggccctgactcttgttaagttaccaatccaggagtgtggtacttgactgtgcaggttgag-3’

Homologous Arm EGFP cDNA TdTomato cDNA Splicing Acceptor LoxP LoxP2272

26 bp sequence (5’-ggagcatgcagctttttcttgggttt-3’)were deleted to destroy sgRNA target.
